# Making choice between competing rewards in uncertain vs. safe social environment: role of neuronal nicotinic receptors of acetylcholine

**DOI:** 10.3389/fnhum.2013.00468

**Published:** 2013-08-27

**Authors:** Jonathan Chabout, Arnaud Cressant, Xian Hu, Jean-Marc Edeline, Sylvie Granon

**Affiliations:** Centre de Neuroscience Paris Sud, Centre National de la Recherche Scientifique UMR 8195, Université Paris Sud 11Orsay, France

**Keywords:** decision-making, social interaction, mice, beta2 nAChRs, ultrasonic vocalization, uncertainty

## Abstract

In social environments, choosing between multiple rewards is modulated by the uncertainty of the situation. Here, we compared how mice interact with a conspecific and how they use acoustic communication during this interaction in a three chambers task (no social threat was possible) and a Social Interaction Task, SIT (uncertain situation as two mice interact freely). We further manipulated the motivational state of the mice to see how they rank natural rewards such as social contact, food, and novelty seeking. We previously showed that beta2-subunit containing nicotinic receptors-β2^*^nAChRs- are required for establishing reward ranking between social interaction, novelty exploration, and food consumption in social situations with high uncertainty. Knockout mice for β2^*^nAChRs-β2^−/−^mice- exhibit profound impairment in making social flexible choices, as compared to control -WT- mice. Our current data shows that being confronted with a conspecific in a socially safe environment as compared to a more uncertain environment, drastically reduced communication between the two mice, and changed their way to deal with a social conspecific. Furthermore, we demonstrated for the first time, that β2^−/−^ mice had the same motivational ranking than WT mice when placed in a socially safe environment. Therefore, β2^*^nAChRs are not necessary for integrating social information or social rewards *per se*, but are important for making choices, only in a socially uncertain environment. This seems particularly important in the context of Social Neuroscience, as numerous animal models are used to provide novel insights and to test promising novel treatments of human pathologies affecting social and communication processes, among which Autistic spectrum disorders and schizophrenia.

## Introduction

Choosing among different rewards relies on multiple processes such as gaining knowledge about existing rewards and their respective value, integrating our own motivational state for each of them, as well as our individual goals. The ability to establish a rank between different rewards is thus a complex process that allows cognitive flexibility, goal focusing, and appropriate decision-making (Chambers et al., 2007; Körding, 2007; Badre, 2012; Smaldino and Richerson, 2012). In addition, the decision making process is complicated by different kinds of dilemmas, such as the one reflected by the exploitation/exploration process (Sutton and Barto, 1998), with striatal dopaminergic mechanisms being strongly linked to the automaticity of exploitation (Everitt et al., 2008; Maia, 2009). Exploration, by contrast, varies following the uncertainty of the different outcomes in competition and the prefrontal cortex plays a pivotal role in tracking uncertainty levels (Daw and Doya, 2006; Daw et al., 2006; Strauss et al., 2011).

In some environments, such as social ones, choosing between concurrent rewards is highly modulated by the uncertainty of the situation. Indeed, if social contacts constitute a reward for social mammals (Panksepp and Lahvis, 2007; Trezza et al., 2011), they may also trigger unknown reactions from social partners, thus making social environment uncertain and potentially risky.

We previously showed that animals lacking beta2 subunit of neuronal nicotinic receptors (β2^−/−^mice) showed impaired behavioral flexibility and difficulty to switch from one reward to another, whether the switch was between social interaction and food consumption, food retrieval and novelty exploration, or novelty exploration and social contact (Granon et al., 2003; Serreau et al., 2011). Particularly, in a social interaction task (SIT) designed to emphasize free social interaction, with potential risk of aggressiveness by an unknown conspecific (Cambon et al., 2010), we showed that β2^−/−^mice exhibited higher level of dominance and lower level of flexibility, in relation with their prefrontal hyper-monoaminergia (Coura et al., 2013). In addition, using a dedicated software to pinpoint social decisions by the probabilistic analysis of more than 20 social sequences within the normal social repertoire (De Chaumont et al., 2012), we showed that depleting the noradrenergic prefrontal innervation in normal mice shrinks the decision tree in this task, with lesioned mice making more rigid and non-adaptive decisions leading to aggressiveness (Coura et al., 2013). A deeper analysis of β2^−/−^ mice' behavior (De Chaumont et al., 2012) was performed by the off line dissection of their behavioral repertoire during the SIT. We identified one peculiar dual risk-prone posture, called “back-to-back,” that requires the progressive development of tolerance from both mice. Indeed, when in this “back-to-back” posture, both mice of the dyad tolerate to be outside of the field of view of the other mouse. We showed that this specific posture emerged progressively while social contact frequency decreased. We thus postulated that this posture that does not exist at first when animals just met, reflects the tolerance they develop for a novel adult male conspecific.

As β2^−/−^ mice did not integrate their partner's behavioral choices -stop, escape, approach- for adapting their own choices, this risk-prone posture virtually never emerged in β2^−/−^ mice, leading to the continuous reinforcement of a unique motivation (i.e., social contact), instead of a switch between novelty exploration and social reinforcement. It is noticeable that the β2^−/−^mice flexible defect in the SIT was overcome by re-expression of the beta2-containing nAChRs into the prefrontal cortex -PFC- of β2^−/−^mice, thus showing the need for functional cholinergic transmission within the PFC for such integrative processes (Avale et al., 2011).

As we showed that the “pro-social” behavior of β2^−/−^ mice was neither due to an impulsive phenotype nor to a biased evaluation of food or social reward values (Serreau et al., 2011), we wondered, here, whether β2^−/−^ mice exhibited difficulties in dealing with competing rewards when they can make free choice, in a safe environment. Indeed, in previous work (Serreau et al., 2011), we put in a same novel arena a novel conspecific and attractive food. We saw that β2^−/−^ mice disengaged less easily from a reinforcing behavior than WT mice, if reinforcements were in conflict with one another. Also, if WT mice frequently switched from one motivation to the other, the frequency of these transitions were biased in β2^−/−^ mice in favor of social motivation. We particularly observed that β2^−/−^ mice were more ready to discard a food reward if the social conspecific approached them (Serreau et al., 2011). It was therefore unkown whether β2^−/−^ mice were more attracted by the social partner because social rewards were more interesting to them, or if they replied more strongly to a social partner that they may perceive as a putative threat. The latter point could be linked to their major increase in dominance behaviors (Coura et al., 2013), and their proneness to exhibit rigid follow behaviors (De Chaumont et al., 2012).

In the current study, we thus defined two types of environments: a “socially safe” one, represented by the 3-chamber apparatus, in which the test animal did not make real physical contact with the social partner, although it was able to see, smell and hear it. Therefore, there was no physical threat, and the choices made by the test mouse were more likely to rely on its own internal state and motivation. The second type of social environment was a large and novel cage in which a dyad of mice interacted freely. The risk of physical threat and dominance existed, although real aggressiveness was extremely rare in the C57BL/6 strain, in this particular protocol (Coura et al., 2013). We defined this situation as “socially uncertain.” It is noticeable that both environments were novel and that the putative stress induced by novelty was diminished by prior exploration.

Here, we compared the ability of WT and β2^−/−^ mice in comparing different natural rewards two by two -social, food or novelty exploration- in a “safe” environment, the three chambers task (Crawley, 2007; Chadman et al., 2008; Silverman et al., 2010a,b). The particularity of the task is that the test mouse is free to explore each reward, without any threat resulting from another male mouse's direct contact. In addition, we wondered whether mice emit ultrasonic vocalizations—USVs—when they were in contact with non-social rewards (such as food). Indeed, it is known that mice emit USVs in both social or non-social contexts (Panksepp and Lahvis, 2007; Jamain et al., 2008; Scattoni et al., 2008, 2010; Chabout et al., 2012). Our recent work showed that the number of emitted USVs correlates with the duration of social contact, and were strongly modulated by motivational/emotional states (Chabout et al., 2012). Acoustics parameters, like peak frequency, duration and number of calls, were dependent of the behavioral context, with high frequency USVs uttered in social (positive or attractive status) context while low frequency USVs were uttered in restrain (negative status) context. Therefore, the integrated analysis of behavioral and communication data may provide novel insight as to the emotional states of mice when confronted to competing rewards in a safe environment.

The aim of this study consisted in providing answers to three main questions:

1a. In a “safe” situation will WT and β2^−/−^ mice show similar reward ranking?
1b. What are the acoustic parameters that would characterize those situations?
2. When the original ranking is altered by previous food or social restriction, will WT and β2^−/−^ mice be able to adapt?
3. What is the importance of social feedback exerted by a moving conspecific in social behavior and in the emission of USVs?

## Materials and methods

### Ethics statement and animals

The animals were treated according to the ethical standards defined by the Center National de la Recherche Scientifique for animal health and care in strict compliance with the EEC recommendations (*n*°86/609). All efforts were made to minimize animal discomfort and to reduce the number of animals used. We tested 25 β2^−/−^ and 40 C57BL/6J -hereby called WT- male mice, all reared and purchased from Charles Rivers Laboratories France (L'Arbresle Cedex, France). β2^−/−^ mice were originally generated from a 129/Sv ES cell line as described previously (Picciotto et al., 1995) and backcrossed onto the C57BL/6J strain for 20 generations. Because littermates are not available in the breeding facilities and as the number of backcrosses was high, we used C57BL/6J mice as controls.

They were 11–12 weeks old at their arrival and remained housed in a standard rearing facility in collective cages (4, 5 animals per cage) during one week before any experiment. Room ventilation, temperature and humidity were controlled with a 12/12 light-dark cycle (light on at 8:00 am). They received *ad libitum* water (throughout all experiments) and standard chow (quantity depending on the experiment).

For sessions of “three chambers task” experimental mice were placed in individual cages three weeks before the experiment while animals used as social stimuli remained in collective cages.

For the SIT mice were thereafter placed in individual cages 3 weeks before the experiment while visitor animals remained in collective cages. Visitor mice were all male C57BL/6J mice while experimental mice were either β2^−/−^ or C57BL/6J mice.

### Behavioral procedures

**Figure 1:**
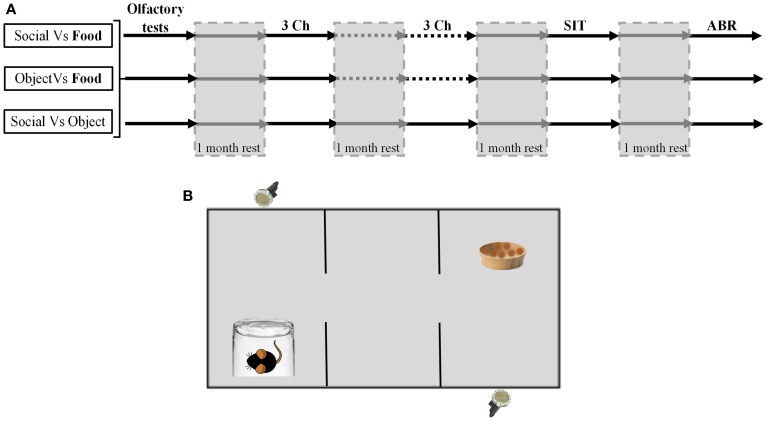
**Experiments design and schematic representation of the three chamber task. (A)** Diagram representing the experiments schedule for the three groups of animals (Social vs. Food, Object vs. Food, Social vs. Object). The gray surfaces corresponded to the rest periods. **(B)** Schematic representation of the three chambers task. Each reward was placed at the opposite of the microphones. Two microphones placed above the cage allowed the recording within only one room.

The succession of behavioral procedures is depicted on Figure 1.

#### Three chambers tasks (3Ch)

Two sessions of 3Ch (respectively, without and with food deprivation) were performed with 1 month interval with the same animals, and by keeping the same group of individuals. The apparatus was a rectangular box (64 × 42 cm) made from translucent Plexiglas. It was divided in three compartments of equal surface by Plexiglas walls. Light was set at 100 Lux and a numeric camera (Hercules®) was placed above the cage allowing to record mouse displacements. We used three different rewards according to the different groups. As a social reward, a naïve C57BL/6J male mouse was placed under a cup. Cups were Plexiglas cylinders with multiple holes to allow breathing, acoustic communication and nose-pokes from both mice. A glass of water was placed on top of the cup to prevent displacement and the test mouse from climbing. Food rewards were sucrose pellets (14 mg, Bio-Serv®), and a cup similar to the one described above was used as a novel object. For the two sessions of 3Ch all mice were habituated to consume sucrose pellets 3 days before the experimental days.

This apparatus allowed us to test mice's preference between two rewards we put in competition. Thus, we used three independent groups, Social *vs.* Food (8 WT, 8 β2^−/−^ mice), Social *vs.* Object (8 WT, 9 β2^−/−^ mice), Food *vs.* Object (8 WT, 8 β2^−/−^ mice). Each group of mice was exposed to only two rewards at a time.

• For the first 3Ch session mice were fed *ad libitum*, with test mice isolated three weeks before the experiment. Tested mice were habituated 10 min to the central room of the apparatus and 10 min to the entire empty apparatus. Social reward mice were habituated to be under the cup for 15 min 3 times per days for 2 days prior experiment.

• The second 3Ch session (after food deprivation) was performed 1 month after the first one. The same groups of animals were maintained during the two sessions. Mice from Social *vs.* Food and Food *vs.* Object groups were deprived of food 2 weeks before the experiment. For deprivation standard chow was given so as to adjust and maintain at 85% of their free feeding weight. Only mice from the Social *vs.* Object condition were not deprived. The latter group allows the control of the repetition effect by comparing the results between the first and the second session of 3Ch in the same mice. Since the mice were tested twice in the same apparatus, we wanted to check that habituation to the maze would not impact the results. Test mice were habituated 10 min to the central room of the apparatus and 10 min to the entire empty apparatus. Social stimuli mice were re-exposed to the cup for 15 min the day before the experiment.

For both 3Ch sessions, after habituation phases, the two rewards were placed in opposite rooms, at the opposite of the microphones side (see Figure 1B). Location (left or right) of each reward was alternated across subject. The test phase lasted 10 min during which video and USVs were recorded. At the end of the experiment, both test and social stimuli mice were replaced in their respective home cage. Between each trial the apparatus was cleaned first with 60% ethanol then with distilled water.

#### Social interaction task (SIT)

The day of the experiment each animal was allowed to visit alone the novel environment for 30 min consisting of a transparent Plexiglas cage containing fresh bedding (50 × 30 × 30 cm) placed in an unfamiliar quiet room. The experimental cage was situated on a table, under a numeric video camera (Hercules®) connected to a computer (recording at 33 frames per s). Light was set at 100 Lux by undirected bulbs. After 30min habituation of the test mouse, a “visitor” mouse was gently introduced into the cage. “Visitors” were male mice unknown from the test mouse, of the same age from the C57BL/6J strain. “Visitors” had always been maintained in social cages. Each dyad was used only once.

#### Parameters of the tasks

In the 3Ch experiment, we scored the time spent and the number of entrances in each reward room, as well as the number and time of contact with each reward. We considered an entrance when the animal placed the two forepaws in one room, and contact with the reward when the animal was less than 1 cm away from the reward. We scored USVs when the animal was in the reward chamber. Therefore, as the time spent in each chamber may vary, we expressed the USVs as the number of calls divided by the time spent in the reward chambers.

In the SIT experiment, we scored manually the duration and number of social contacts and analyzed the behavioral sequences between the two conspecifics for 8 min. Likewise, we scored USVs during the 8 min experiment.

#### Control measures

***Olfaction tests*.** Olfactory tests were devoted to test if mice of both genotypes were able to detect smells (i.e., small volatile molecules carried by the air). These odors are detected by neurons of the main olfactory epithelium. We therefore checked olfactory discrimination between water, orange flavor, and urine of male mice. By contrast, pheromones are detected by a specialized and distinct olfactory system, the vomeronasal organ (Dulac, 2000). As pheromones are present in high concentration in litter, we also subjected mice to a second olfactory test and compared their behavior when confronted to a clean *vs*. a used litter. Both olfactory experiments conducted in 24 WT and 25 β2 KO mice were tested in a transparent cage of Plexiglas (50 × 30 × 30 cm). Their procedures are described below.

*Experiment 1: Comparison between three olfactory stimuli*. This first olfactory experiment was used to test the ability of both groups of mice to discriminate volatile odors. The experiment consisted of 30 min habituation to the cage. During this habituation period, an empty tube was taped to one largest side wall of the cage. The tube consisted of a Pasteur pipette (of which the tip was broken off) with a piece of filter paper (2 × 2 cm) rolled into it. It was taped onto the wall of the cage with a distance of 9 cm between the tip of the pipette and the bottom of the cage. This habituation period was followed by three times 2 min exposure to water, orange and urine odors, successively inserted in the tube. The orange consisted of a 1% solution of natural orange flavor in water. The urine sample was collected from groups of social C57BL6/J male mice. After collection the urine was kept in 0.5 ml Eppendorf tubes and frozen until use. A 20 μl drop of the odor sample was added to the filter before the exposure.

We measured the time spent sniffing the tip of the pipette thanks to *off line* video analyses. The sniffing area was defined by a 2 cm diameter circle around the tip.

*Experiment 2: Fresh litter vs. used litter*. Litter taken from social cages -used litter- which may contain some volatile compounds but that contains mostly non-volatile ones (pheromones), was used to test mice's sensitivity to non-volatile odors components. Two Petri dishes (diameter 10 cm) were taped on the cage's floor. The floor was divided into eight equal square pieces by a piece of paper placed under the cage. Petri dishes contained fresh or used litter (mixture from four different cages of six same genotype male mice). Right or left position of each dish was randomized between each trial. The tested mouse was placed at the center of the cage and freely explored the environment during 15 min. The experimental cage was situated on a table, under a numeric video camera (Hercules®) connected to a computer (recording at 33 frames per s). Light was set at 100 Lux by undirected bulbs.

The time spent digging in each dish, number of exploration moves into the litter (front paws in the cup), number of rearing and grooming were measured and analyzed *off line* on the videos.

***Auditory tests*.** Thresholds for the averaged Auditory Brainstem Response (ABR) were used as an electrophysiological measure of auditory sensitivity (Willott and Erway, 1998; Willott, 2006). These measures were made at the end, after all the behavioral procedures above. For this, calibrated stimuli were delivered using speaker equipment manufactured by DELTAMED. A maximum sound pressure level (SPL re: 20 WPa) of 80 dB was employed for all stimuli. Mice were anesthetized with mixed Xylazine (10 mg/Kg) and Ketamine (150 mg/Kg). Sub-dermal needle electrodes were inserted at the vertex (active), ventrolaterally to the left ear (reference) and in a paw muscle (ground). Mice were tested with tone pips (100 μs rise/fall; 10 ms duration; 1, 2, 4, 5, 8, 12, 16, 24, and 32 kHz). ABR thresholds were obtained for each frequency by reducing the SPL at 10 dB steps and finally at 5 dB steps up and down to identify the lowest level at which an ABR could be recognized. All records were computerized by software; Centor USB, DELTAMED.

#### Ultrasonic vocalization recording

In all experiments, except olfaction tests, a condenser ultrasound microphone Polaroid/CMPA was placed above the experimental chamber, high enough so that the receiving angle of the microphone covered the whole area of the test cage. For the 3Ch condition, one microphone was placed above each side chamber with an angle allowing full chamber coverage but avoiding any recording from the opposite chamber (Figure 1). Microphones were connected to an ultrasound recording interface Ultrasound Gate 416 H, which was itself plugged into a personal computer equipped with the recording software Avisoft Recorder USG (Sampling frequency: 250 kHz; FFT-length: 1024 points; 16-bits). All recording hardware and software were from Avisoft Bioacoustics® (Berlin, Germany).

#### Acoustic variables

For all behavioral conditions USVs were analyzed off line with SASLab Pro (Avisoft Bioacoustic®, Berlin, Germany). Spectrograms were generated for each detected call (Sampling frequency: 250 kHz; FFT-length: 1024 points; 16-bit; Blackman window; overlap: 87.5%; time resolution: 0.512 ms; frequency resolution: 244 Hz). For SIT condition audio recordings were disturbed by the background noise originating from the animals moving and/or digging in the fresh bedding. We nevertheless kept the bedding because social interactions may have been affected by its absence and we wanted to match as closely as possible to our classical experimental conditions (Granon et al., 2003). However, this prevented an automatic analysis of acoustic data.

We recorded the total number of calls emitted by each pair of mice, and manually measured different variables related to peak frequency [*Pf*_start_ (peak frequency at the beginning of the call), *Pf*_end_ (peak frequency at the end of the call), *P*f_min_ (minimum peak frequency), *Pf*_max_ (maximum peak frequency)] for each call allowing us to calculate the *Pf*_mean_ as *Pf*_mean_ = (*Pf*_min_ + *Pf*_max_)/2.

#### Synchronization of audio and video files

We performed a “clap” with our fingers in the field of the camera to time-matched video and audio files. In the audio files, we cut the information before this sound and in the video files we selected the exact frame of this event and started from this point. This manual synchronization allowed us to analyze which USVs were emitted during contact and non-contact events for SIT condition, and which USVs were emitted when test mouse was actually present in the related reward room in the 3Ch.

### Statistical analyses

Statistical analyses were made with Statview® software. ANOVA—repeated measures were used to compare the reward factors two by two (Social vs. Food, Social vs. Object, and Food vs. Object). Repeated measures ANOVA were used to compare subject performances. *Post-hoc* analyses were performed using Wilcoxon signed-rank (for dependent variables) or Mann-Whitney (for independent variables) non parametric tests only when appropriate. Correlation data were analyzed with a Spearman correlation test between behavioral measures and number of calls. The significance threshold was set at *p* < 0.05. For all *post-hoc* paired comparisons a Bonferroni correction was applied (α = α/k; where α is significance threshold and *k* the number of comparisons).

## Results

### Reward ranking of β2^−/−^ mice in safe environment

**Figure 2:**
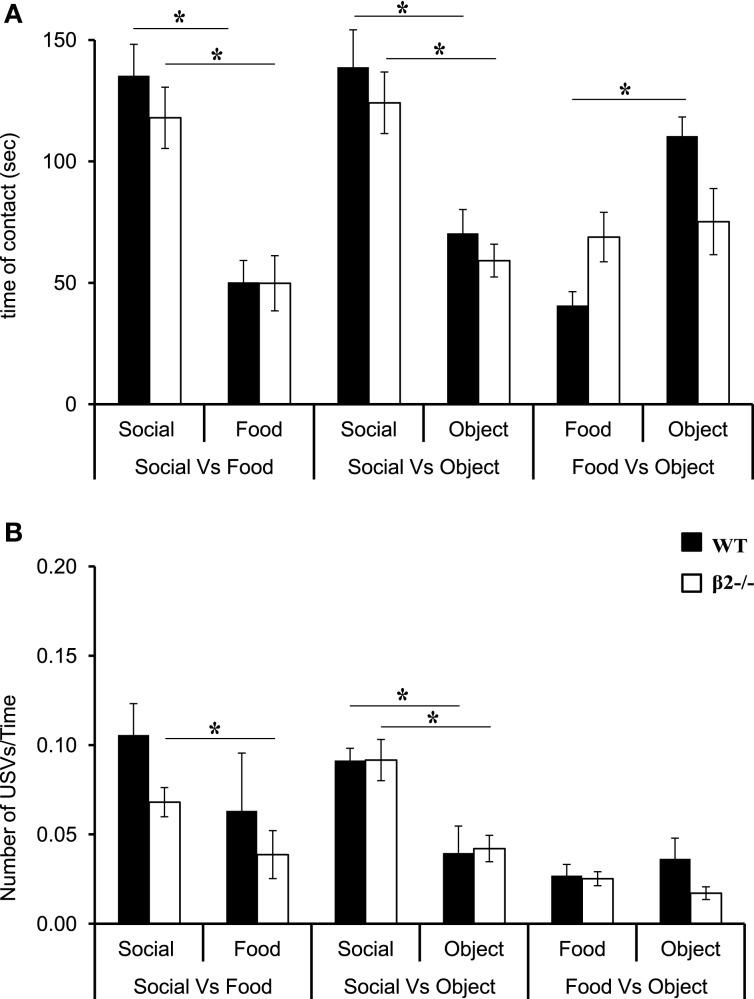
**Reward ranking of β2^−/−^ and WT mice in safe environment. (A)** Time spent in contact with each reward. **(B)** Number of ultrasonic vocalizations— USVs—emitted in contact with each reward divided per the time spent in the room. Data are presented for WT and β2^−/−^ mice as mean ± SE. ^*^p < 0.005; for Mann-Whitney paired comparisons.

We first analyzed contact time in all the non-deprived conditions (Social vs. Food, Social vs. Object, Food vs. Object). For all conditions, there was a major reward effect [S vs. F: *F*_(1, 15)_ = 42.38, *P* < 0.0001; S vs. O: *F*_(1, 14)_ = 32.85, *P* < 0.0001; F vs. O: *F*_(1, 14)_ = 11.18, *P* = 0.0048] and no genotype effect [S vs. F: *F*_(1, 15)_ = 0.75, *P* = NS; S vs. O: *F*_(1, 14)_ = 1.65, *P* = NS; F vs. O: *F*_(1, 14)_ = 0.325, *P* = NS]. There was an interaction only in the Food vs. Object condition [interaction genotype × condition: *F*_(1, 14)_ = 7.7, *P* = 0.01]. A more detailed comparison between rewards revealed that WT and β2^−/−^ mice stayed longer in contact with the social reward, then with the Food [Figure 2A and Table 1, WT: S vs. *F*: *z* = − 2.38, *P* = 0.017; β2^−/−^: S vs. F: *z* = − 2.54, *P* = 0.011], but that they also prefer the Social as compared to a novel object (Figure 2A and Table 1, WT: S vs. O: *z* = −2.38, *P* = 0.017; β2^−/−^: S vs. O: *z* = −2.38, *P* = 0.017). However, β2^−/−^ mice spent similar time in contact with the food and the novel object in the Food vs. Object condition, while WTs spent more time in contact with the novel object (Figure 2A and Table 1, WT: F vs. O: *z* = −2.52, *P* = 0.011; β2^−/−^: F vs. O: *z* = −0.42, *P* = 0.67). We noticed that the number of entrance in each compartment (data not show) was similar in both genotypes and for all the conditions. Therefore, even if β2^−/−^ mice are hyperactive (Granon et al., 2003), this cannot explain the difference between the two genotypes concerning the time spent in contact with each reward.

**Table 1:** **Summary of the reward ranking according to the two exposures of the three chambers tasks, before food deprivation and after food deprivation, for WT and β2^−/−^ mice**.

	**Non-deprived condition**	
	**Time in contact**	**Number of USVs**
WT	Social > Food	Social = Food
	Social > Object	Social > Object
	Object > Food	Food = Object
β2^−/−^	Social > Object	Social > Food
	Social > Object	Social > Object
	Food = Object	Food = Object
	**Deprived condition**	
	**Time in contact**	**Number of USVs**
WT	Food > Social	Social > Food
	Food > Object	Food = Object
β2^−/−^	Food > Social	Social > Food
	Food > Object	Food = Object

When mice were in contact with rewards they emitted USVs. The number of calls was dependent of the time spent in each compartment (if they spent more time in the food compartment the probability to emit calls was higher). To circumvent this bias, we calculated the ratio of the number of USVs divided by the time spent in a given compartment. Results showed that when mice were not food deprived, such as in Social vs. Food and Social vs. Object conditions, there was a reward effect [S vs. F: *F*_(1, 15)_ = 9.94, *P* = 0.006; S vs. O: *F*_(1, 15)_ = 25.59, *p* = 0.0002], but no genotype effect [S vs. F: *F*_(1, 15)_ = 1.70, *p* = NS; S vs. O: *F*_(1, 15)_ = 0.019, *p* = NS] and no interaction reward × genotype (S vs. F: *p* = NS, S vs. O: *p* = NS). We showed that WT and β2^−/−^ mice always emitted more USVs in contact with social rewards than in contact with object rewards, while only β2^−/−^ emitted more USVs in contact with social rewards in the Social vs. Food condition (Figure 2B and Table 1, WT: S vs. *F*: *z* = –1.68, *p* = 0.09; S vs. O: *z* = –2.1, *p* = 0.03; β2^−/−^: S vs. F: *z* = –2.19, *p* = 0.02; S vs. O: *z* = –2.38, *p* = 0.01). We showed that there was no difference between USVs uttered in Food vs. Object condition for both genotypes [Reward effect: *F*_(1, 15)_ = 0.019, *p* = NS; genotype effect: *F*_(1, 15)_ = 1.72, *p* = NS].

Furthermore, we analyzed the peak frequency mean, Pf mean, of these calls in each compartment. Interestingly, WT and β2^−/−^ showed no differences (not shown). However, the Pf mean was lower in contact with social reward for both genotypes (WT: 51.9 ± 1.6 kHz; β2^−/−^: 48.7 ± 1.4 kHz) than Pf mean in food reward (WT: 61.4 ± 2.8 kHz; β2^−/−^: 64 ± 4.3 kHz) as well as in contact with the object reward (WT: 61.1 ± 2.6 kHz; β2^−/−^: 60.2 ± 2.8 kHz). This result led us to think that the social mouse placed under the cup, although habituated to the procedure, contributed to the low frequency calls.

### Adaptive behavior of β2^−/−^ mice when motivational state changes

In the second sessions of 3Ch task, all animals were food deprived except for the Social vs. Object group. As animals didn't need to be deprived (no food involved), this condition allowed us to control the repetition effect between the first and the second 3Ch exposures. There was no repetition effect between the first and the second Social vs. Object experiment for the time in contact with rewards [Social: repetition effect: *F*_(1, 14)_ = 1.364, NS; genotype effect: *F*_(1, 14)_ = 0.194, NS, Object: repetition effect: *F*_(1, 14)_ = 1.859, NS; genotype effect: *F*_(1, 14)_ = 3.30, NS].

**Figure 3:**
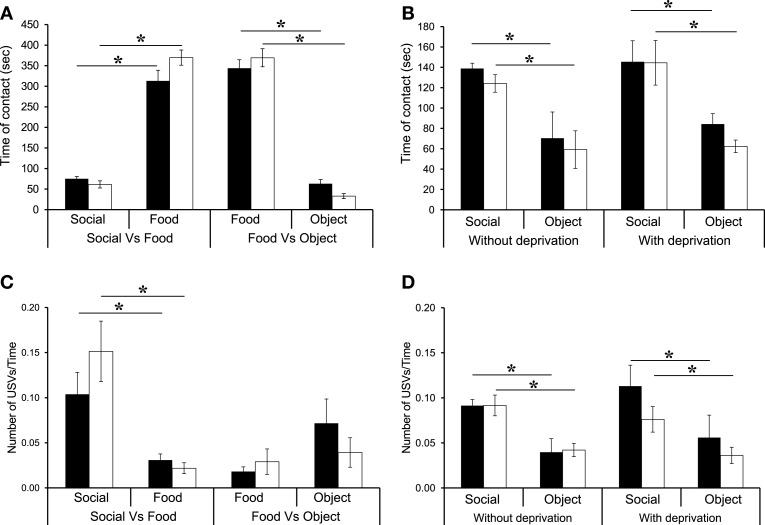
**Reward ranking of β2^−/−^ and WT mice after food deprivation. (A)** Time spent in contact with each reward. **(B)** Number of entrance in each room containing the rewards. **(C)** Number of ultrasonic vocalizations—USVs—emitted in contact with each reward divided per the time spent in the room. **(D)** Reduction of Pf mean observed between the first session (without deprivation) and the second session (food deprivation) of the 3Ch task. Datas are presented for WT and β2^−/−^ mice as mean ± SE. ^*^p < 0.005; for Mann-Whitney paired comparisons.

In all conditions (Social vs. Food, Food vs. Object), when the motivational state changed after food deprivation, there was no difference between WT and β2^−/−^ mice [genotype effect: S vs. F: *F*_(1, 14)_ = 2.31, NS; F vs. O: *F*_(1, 14)_ = 0.043, NS], but there was a reward effect [S vs. F: *F*_(1, 15)_ = 289.61, *p* < 0.0001; F vs. O: *F*_(1, 14)_ = 287.65, *p* < 0.0001]. As expected, WT and β2^−/−^ mice spent most of their time in contact with the Food reward as compared with the Social reward (Figure 3A and Table 1, WT: *z* = −2.36, *p* = 0.01; β2^−/−^: *z* = −2.66, *p* = 0.007), or with Object rewards (Figure 3A and Table 1, WT: *z* = -2.52, *p* = 0.011; β2^−/−^: *z* = −2.38, *p* = 0.01). In addition, we showed that both WT and β2^−/−^ mice spent more time in contact with social than object rewards during the second session of three chamber task [Figure 3B right panel, genotype effect: *F*_(1, 14)_ = 0.74, *p* = NS, Reward effect: *F*_(1, 14)_ = 32.84, *p* < 0.0001; WT: *z* = −2.52, *p* = 0.011, β2^−/−^: *z* = −2.38, *p* = 0.017].

As previously, we showed that there was no difference in the number of entries in each compartment (data not shown). These results showed that even if mice spent more time in contact with the food rewards, they did not neglect the other rewards.

Regarding the emission of calls, only the Social vs. Food (Figure 3C) and the Social vs. Object conditions (Figure 3D, right panel) showed a reward effect when animals were food deprived [S vs. F: *F*_(1, 14)_ = 26.81, *p* = 0.0001, S vs. O: *F*_(1, 14)_ = 22.17, *p* = 0.0003] but no genotype effect [*F*_(1, 14)_ = 2.21, NS] or interaction reward × genotype [S vs. F: *F*_(1, 14)_ = 1.91, NS, S vs. O: *F*_(1, 14)_ = 0.53, NS]. The amount of calls emitted in the Food vs. object condition was similar for both genotypes for both rewards, even if WT mice showed a marginally significant trend to emit more USVs when in contact with the novel object than with food (F vs. O: WT: *z* = −1.85, *p* = 0.06). Actually in WT and β2^−/−^ mice, the ratio USVs/Time in contact was always higher with social rewards than with food rewards (S vs. F: WT: *z* = −2.36, *p* = 0.01; β2^−/−^: *z* = −2.66, *p* = 0.007; S vs. O: WT: *z* = −2.19, *p* = 0.02; β2^−/−^: *z* = −2.54, *p* = 0.01).

We observed that food deprivation altered some USV features, like peak frequency and duration of calls. In both WT and β2^−/−^ mice there was a significant reduction between non-deprived and deprived conditions in the mean peak frequency for both Social vs. Food [data not shown, Social: condition effect: *F*_(1, 15)_ = 17.47, *p* = 0.0009, genotype effect: *F*_(1, 14)_ = 3.40, NS, interaction condition × genotype: *F*_(1, 15)_ = 0.017, NS; Food: condition effect: *F*_(1, 13)_ = 9.05, *p* = 0.01, genotype effect: *F*_(1, 13)_ = 4.12, *p* = NS, interaction condition × genotype: *F*_(1, 13)_ = 0.28, NS], and Food vs. Object conditions [data not shown, Food: condition effect: *F*_(1, 14)_ = 5.33, *p* = 0.03, genotype effect: *F*_(1, 14)_ = 2.49, *p* = NS, interaction condition × genotype: *F*_(1, 14)_ = 0.41, NS; Object: condition effect: *F*_(1, 13)_ = 1.28, *p* = 0.27, genotype effect: *F*_(1, 13)_ = 0.44, NS, interaction condition × genotype: *F*_(1, 13)_ = 1.08, NS]. Indeed, during Social vs. food, WT mice showed a significant reduction of Pf mean (15.79%) when in contact with the social reward (*U* = 7, *p* = 0.02), but not with the food reward (*U* = 15, NS). In addition, β2^−/−^ mice showed a significant reduction of Pf mean (16.33%) when in contact with the social (*U* = 14, *p* = 0.01) and with the food rewards (16.67%; *U* = 14, *p* = 0.0.3). In the Food vs. Object condition, both WT and β2^−/−^ mice showed a significant decrease in Pf mean when in contact with the Food reward (respectively 12.84% *U* = 11, *p* = 0.02, 19.96% *U* = 9, *p* = 0.01). Neither WT nor β2^−/−^ mice showed such a decrease when in contact with the object reward (WT: *U* = 27, NS; β2^−/−^: *U* = 12, NS).

### Importance of social feedback

**Figure 4:**
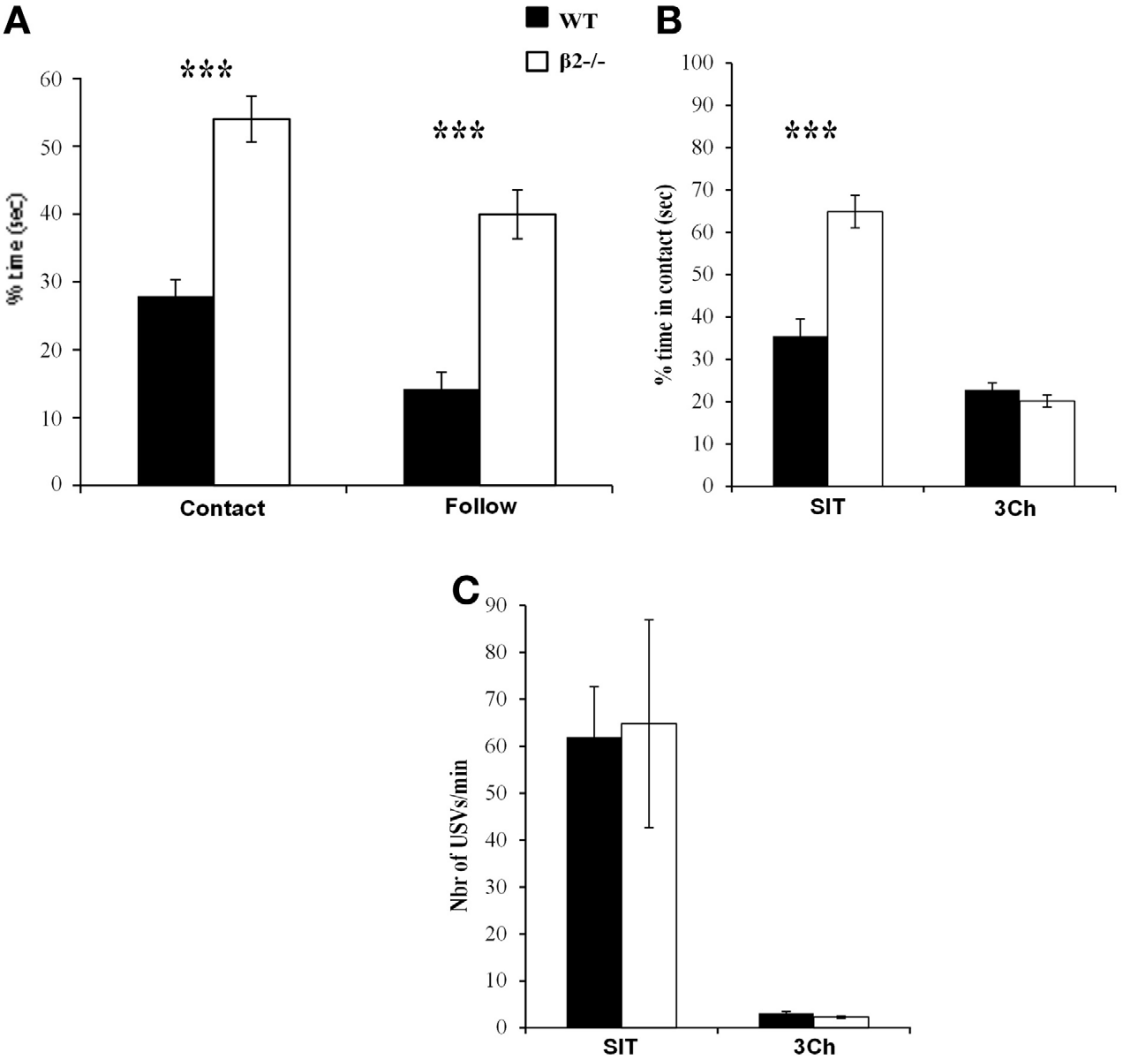
**Effect of social feedback. (A)** Percentage of time spent in contact to, and in following the conspecific during the social interaction task (SIT). **(B)** Percentage of time spent in contact with the conspecific in the social interaction task (SIT) and in the 3Ch task (3Ch). **(C)** Number of ultrasonic vocalizations—USVs—emitted in each condition. Datas are presented for WT and β2^−/−^ mice as mean ± SE. ^***^p < 0.0001 for Mann-Whitney paired comparisons.

When mice were tested in SIT 1 month after the last session of 3Ch, we observed the typical phenotype of β2^−/−^ mice (Granon et al., 2003; Avale et al., 2011; Serreau et al., 2011; De Chaumont et al., 2012). β2^−/−^ mice spent more time in contact with the conspecifics than WT mice (Figure 4A,*U* = 24.5, *p* < 0.0001) and showed increased follow behaviors (Figure 4A, *U* = 28, *p* < 0.0001).

We wanted to know why we observed little, if any, difference in the 3Ch task while in SIT, WT and β2^−/−^ mice behave very differently. Thus, we directly compared WT and β2^−/−^ mice behavior and USVs in these two social conditions that trigger different “levels” of social reward. Indeed, the SIT provides full social contact (physical contact, movements of both mice, visual, olfactory and auditory feedbacks) while the three chamber task provides only limited amount of social information (visual, olfactory and auditory, with contact limited to nose pokes). We showed that the level of social information impacted the time spent in contact with social reward [Figure 4B, condition effect: *F*_(1, 31)_ = 111.2, *p* < 0.0001]. Indeed, both WT and β2^−/−^ mice spent more time in social contact during the SIT than during the 3Ch task (WT: *z* = −2.79, *p* = 0.0052; β2^−/−^: *z* = −3.62, *p* = 0.0003).

Like observed in our previous experiments, β2^−/−^ mice spent significantly more time in contact with the conspecific than WT mice in SIT (*U* = 24.5, *p* < 0.0001), but not in the 3Ch (*U* = 100, NS), as illustrated in Figure 4.

**Figure 5:**
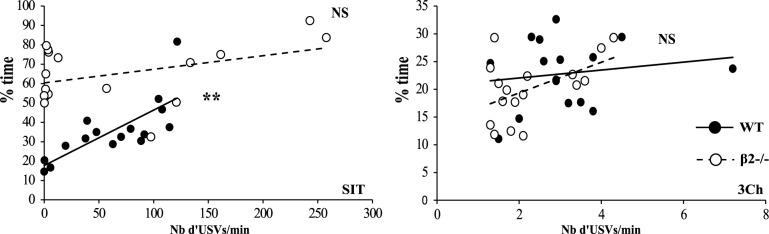
**Correlation between time in social contact and emission of USVs.** Correlation between the number of calls and duration of contact in the SIT and 3Ch tasks. Datas are presented for WT and β2^−/−^ mice. ^**^p = 0.005 for Spearman rank correlation test and NS: *p* > 0.05.

We also analyzed USVs emitted during both SIT and 3Ch conditions (Figure 4C). We showed that there was no difference between WT and β2^−/−^ mice but call rate (number of USVs per min) varied between conditions [genotype effect, *F*_(1, 31)_ = 0.007, *p* = 0.93, NS; condition effect, *F*_(1, 31)_ = 24.62, *p* < 0.0001, interaction genotype × condition, *F*_(1, 31)_ = 0.023, NS]. WT and β2^−/−^ mice emitted drastically more USVs during the SIT (WT: 61.9 ± 10.76, β2^−/−^: 64.77 ± 22.13 USVs per min) than during the 3Ch task (WT: 3.11 ± 0.35, β2^−/−^: 2.28 ± 0.25 USVs per min). Furthermore, in the SIT, there was a positive and significant correlation between the time in social contact and the number of USVs emitted for WT (Figure 5), (*r*s = 0.821, *n* = 16, *p* = 0.0015), but not for β2^−/−^ mice (*r*s = 0.434, *n* = 17, NS). There was no such correlation in the 3Ch task for any genotype (WT: *r*s = 0.099, *n* = 16, NS; β2^−/−^: *r*s = 0.314, *n* = 17, NS).

### Control measures

#### Olfactory tests

***Experiment 1*.** β2^−/−^ mice spent significantly less time sniffing odors, whatever it was than WT mice [genotype effect: *F*_(1, 47)_ = 21.32, *P* < 0.0001]. For both genotypes no differences were detected between global time spent sniffing water, orange or urine [condition effect: *F*_(2, 47)_ = 1.49, *P* = NS; interaction genotype × condition: *F*_(2, 94)_ = 0.647, *P* = NS, data not shown]. However, when comparing the last exposure to water and the first exposure to orange, both WT and β2^−/−^ mice reacted to the change (parametric *t*-test *p* = 0.056 and *p* = 0.028, respectively). When comparing the last exposure to orange and the first exposure to urine, only WT mice reacted to the change (parametric *t*-test *p* = 0.046), while β2^−/−^ mice did not show significant difference (*p* = 0.1), likely because of a large inter-individual variability which could be associated with their hyperactive phenotype.

***Experiment 2*.** The second olfactory test was to check the interest of β2^−/−^ mice in social odors such as pheromones. We compared their behavior when exposed to litter taken from social cages vs. clean litter. There was no difference between WT and β2^−/−^ [genotype effect: *F*_(1, 47)_ = 0.042, *p* = NS], and both genotypes spent drastically more time in contact with social than with clean litter [data not shown, condition effect: *F*_(1, 47)_ = 156.03, *P* < 0.0001; interaction genotype × condition: *F*_(1, 47)_ = 2.05, *p* = NS]. These control experiments showed that β2^−/−^ mice exhibit similar interest for social olfactory cues as WT animals.

**Figure 6:**
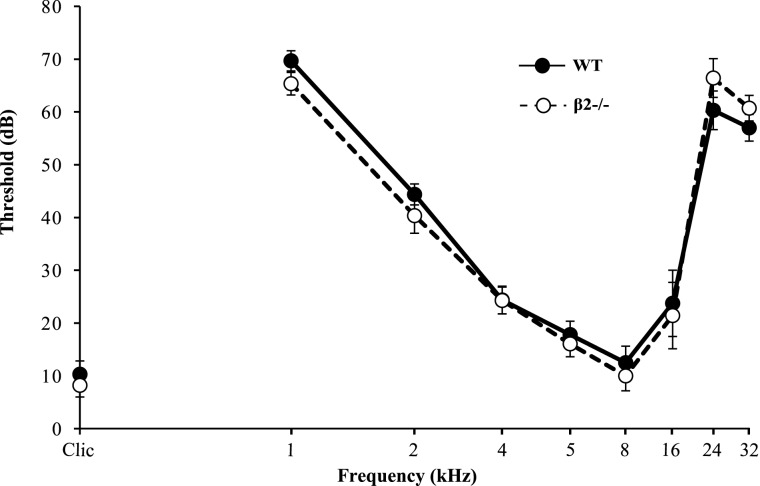
**Auditory brainstem responses (ABR) elicited by WT and β2^−/−^ mice.** Graph showed the auditory brainstem responses for WT and β2^−/−^ mice for different pure frequency sounds (logarithmical scale) at different thresholds (dB).

To control for putative auditory defects in β2^−/−^ mice, we analyzed their ABR. Results showed no difference between WT and β2^−/−^ mice [*F*_(1, 28)_ = 0.139, NS] and both genotypes exhibited auditory thresholds that were function of the tone frequency [*F*_(8, 28)_ = 115.13, *p* < 0.0001] and that were similar to previously published ABR thresholds (Buran et al., 2010). Therefore, the lack of correlation between the number of USVs and the time spent in social contact during the SIT in β2^−/−^ mice is unlikely to be due to auditory problems (Figure 6).

## Discussion

The aim of the present study was to determine whether, and if yes how, mice rank natural rewards like food, exploration and social contact. In addition, we wondered whether being in the 3Ch task, i.e. a task in which the test mouse can make choices without interference from another mouse, would impact on this rank and how social information were integrated to choose between rewards. We further asked whether β 2nAChRs, known to be necessary for showing adapted social interaction, would be involved in such reward ranking.

We focused here on three main natural rewards in rodents: novelty exploration, interaction with an unkown conspecific, and food consumption. By contrast with our previous studies, we used a three chamber task (3Ch task) to assess the rank spontaneously established by mice between natural rewards in a safe environment. Indeed, in the 3Ch task these rewards competed two by two, and mice can freely move from one reward to another without any interference from a conspecific. We then modified food motivation by food deprivation, and assessed the ability of mice to adapt to their motivational state. In both situations, we compared behaviors and ultrasonic vocalizations.

Previous works showed that when these three rewards competed in socially unpredictable environment like SIT, β2^−/−^ mice showed impaired organization in their choices toward the rewards, namely, they exhibited difficulty in switching between the different rewards (Granon et al., 2003; Serreau et al., 2011). In such environment, the tested mouse faced an unknown conspecific -the visitor mouse- which moved freely and showed reciprocal and non-aggressive social contact. We further showed that β2^−/−^ mice exhibited decision-making defects and lacked behavioral flexibility, whether the rewards were of social nature (De Chaumont et al., 2012), or not (Granon et al., 2003). However, in the SIT context, the visitor mouse strongly interacted with the test mouse, thus potentially affecting its decisions. β2^−/−^ mice also exhibited a high level of dominance toward the visitor mouse (Coura et al., 2013) and were less likely to allow the visitor mouse approaching (Serreau et al., 2011; De Chaumont et al., 2012). To circumvent this issue, we used here a safe and predictable environment, the three chambers environment (Crawley, 2000; Moy et al., 2004; Nadler et al., 2004; Silverman et al., 2010a). In this task, the test mouse (either a WT or a β2^−/−^ mouse) was the only decision-maker, as the stimulus mouse was kept under a cup during “Social” reward sessions. This test therefore allowed us to establish the natural preference exhibited by the tested mice. In this context, our current results show that the rank established between rewards was adaptive for both genotypes: it changed similarly in WT and β2^−/−^ mice when the motivation level of mice changed, i.e., when animals were food deprived. As compared to a socially more unpredictable environment (Serreau et al., 2011), the 3Ch experiment revealed that the establishment of a rank between competing motivations was strongly modulated by the social risk level of the task, or by the putative interference from an unkown adult male mouse. Indeed, we show here that non food-deprived WT mice ranked their motivations in a specific order, from the most preferred reward to the less preferred one: social > novel object > food. This result confirmed that novelty exploration is one of the preferred natural rewards in mice (Avale et al., 2011; Bourgeois et al., 2012). Whether this was reinforced by the paucity of the laboratory rearing in standard cages, i.e., containing no items, remains to be investigated (Van Praag et al., 2000; Kulesskaya et al., 2011). It is noticeable that scoring the number of entrance in a specific compartment was not sufficient as this measure did not allow discrimination between the different rewards, contrary to the scoring of duration of contact with each reward.

Our results revealed that non-food deprived β2^−/−^ mice, like WT mice, chose the social reward in the first place. However, they spent equal time in contact with the novel object and the food. As we previously showed that β2^−/−^ mice are not more -or less- sensitive to food reward than WT (Serreau et al., 2011), the current data may suggest that for β2^−/−^ mice, food can be considered as an interesting novel object, when the food motivation is low.

The number of USVs emitted was significantly higher when mice faced the social reward than when they faced any of the two other rewards. This was true for both WT and β2^−/−^ mice. If this measure obviously increased when having two mice instead of one, it also confirmed the stimulating effect of the social context on USV's emission (Vignal et al., 2005; Arriaga et al., 2012; Chabout et al., 2012). It is interesting to note that having two mice instead of one did not simply multiply by two the number of USVs. Indeed, comparing the number of USVs in the 3Ch (social condition) and in the SIT, two experimental conditions in which a dyad of mice was recorded, clearly showed that the number of animals was not a critical factor. By contrast, the type of social contact they can have was likely to be a major factor. Indeed, we showed here that in the 3Ch task, the distribution of USVs was statistically not correlated to the time spent in contact with the rewards. This was not the case in the SIT during which both mice exchanged not only olfactory, auditory and visual information but also could touch and react to each other. Our ABR and olfactory control experiments showed that it is unlikely that differences between WT and β2^−/−^ mice were due to difference in integrating olfactory or auditory information, although it must be noticed that ABR did not measure auditory responses for the highest USVs.

The behavioral results also showed that in the 3Ch environment, both genotypes reacted similarly to the food deprivation and re-organized their reward ranking when their motivation for food changed: they both decreased the time spent in contact with social reward or with the novel object, and increased drastically the time spent in contact with the food, as expected. This showed that both groups were similarly sensitive to food deprivation and adapted their reward preference to their motivation level. However, the number of USVs emitted in contact of each reward was similar in food deprived and non-deprived conditions. Both β2^−/−^ and WT mice emitted more USVs in the social compartment. However, both groups showed similar alteration in the mean frequency of emitted USVs (about 5–10% lower) after food deprivation. It has been shown that when rats (Brudzynski, 2007) or mice (Chabout et al., 2012) were subjected to a negative emotional context, such as foot shock of restrain stress exposure, the frequency of their USVs was lower than when they were exposed to a positive or rewarding stimulus. Our current results therefore suggest that food deprivation induced a slightly negative emotional state that was reflected by the frequency of the USVs emitted. However, USVs thus emitted did not discriminate between the different rewards, suggesting that the lower emotional state induced by food deprivation was not counterbalanced by other types of rewards.

Notably, β2^−/−^ mice exposed to the 3Ch task showed no difference with WT mice concerning their behavior and USVs. However, when these animals were subsequently subjected to the SIT, they exhibited a drastic behavioral impairment: they specifically showed increased social contact duration and follow behavior. These results, that are similar to our previously data obtained in the SIT in animals that were not exposed to other tasks before (Granon et al., 2003; Avale et al., 2008; De Chaumont et al., 2012; Coura et al., 2013), highlight two important points. First, although β2^−/−^ mice exhibited normal motivation for social reward when tested in the 3Ch, they showed altered social interaction when the social environment was unpredictable. Second, our results emphasized the importance of social feedback. We showed that in the 3Ch task, the number of USVs was drastically and significantly reduced, as compared to that emitted during the SIT. Furthermore, we showed that in WT mice, this number was not correlated to the duration of social contact, although this was the case in the SIT. A large part of USV emission in the SIT was therefore likely to be associated with the social feedback received by the dyad. Another alternative hypothesis, not exclusive with the first one, is that USVs accompanied the attentional load generated by the task. This load would be higher in unpredictable tasks and so would be the number of USVs.

The lack of correlation between the duration of social contact and USVs in β2^−/−^ mice subjected to the SIT could mean that these mice were not sensitive to the social reward. However, results obtained in the 3Ch task indicate that this is unlikely. Rather, the lack of correlation may indicate that β2^−/−^ mice did not integrate USVs in social behavior.

Our current data revealed that the 3Ch task and the SIT are both very complementary in the study of mice social interaction. The 3Ch task is very useful to ensure that animals exhibit normal preference for a social conspecific, as compared to other types of rewards. It can also be used to show that animals exhibit normal social approach or interest. However, the fact that very few USVs were obtained in this task limits its use. The SIT, by contrast, and because it allows social feedback from both conspecifics, can be used for studying behavioral social patterns and strategies as well as how acoustic communication is integrated in these patterns. Placing animals in such environment, although it remains quite different from a naturalistic context, allows to study how mice face risk and, potentially threat, from another unknown individual, or develop dominance. Using the SIT, we previously showed that re-expressing the beta2-containing nAChRs into the prefrontal cortex of β2^−/−^ mice was sufficient to restore normal pattern of social interaction (Avale et al., 2011). Whether this behavioral restoration would be associated with restoration of USV-social contact duration correlation and whether this correlation is necessary for the restoration of social flexibility remains to be unraveled.

What determines social behaviors remains unclear. However, the fact that they were conserved during evolution process and shared by most animal species suggests that there are great benefits to them. Here, although we did not compare the three rewards at the same time, we demonstrated that mice establish a rank among competing natural rewards. Social reward was the preferred one, if mice have been socially deprived for a few weeks. We also provide clear evidence that β 2-containing nAChRs are not involved in motivational ranking *per se*, as β2^−/−^ mice showed normal reward ranking in a safe social situation. These receptors were also not involved in the monitoring of internal states as β2^−/−^ mice adapted, like WT mice to food or social deprivation. We also highlight that social feedback and acoustic communication are related. It remains unclear, however, if social feedback impacts communicational abilities or, in the contrary, if alteration of USV features impact social behaviors.

## Author contribution

Jonathan Chabout and Arnaud Cressant analyzed the data, performed the statistical analyses. Jonathan Chabout carried out the behavioral experiments and participated in the writing process. Xian Hu and Arnaud Cressant carried out some behavioral experiments. Jean-Marc Edeline and Sylvie Granon contributed to interpretation and writing process. Sylvie Granon designed, contributed, and coordinated the experimental work.

### Conflict of interest statement

The authors declare that the research was conducted in the absence of any commercial or financial relationships that could be construed as a potential conflict of interest.
